# Identifying the Diagnostic Challenges and Indicators of Orthostatic Tremor: Patient Perspectives

**DOI:** 10.1002/mdc3.70081

**Published:** 2025-04-23

**Authors:** Wietske A. Babeliowsky, Kiel Woodward, Bart Swinnen, Sarah Doss, Michael Muir, Rob M.A. de Bie, Diego R. Torres‐Russotto, Anne‐Fleur van Rootselaar

**Affiliations:** ^1^ Department of Neurology and Clinical Neurophysiology Amsterdam University Medical Centers, Amsterdam Neuroscience, University of Amsterdam Amsterdam The Netherlands; ^2^ Movement Disorders Section, Department of Neurological Sciences University of Nebraska Medical Center Omaha Nebraska USA; ^3^ Department of Neurology University Hospitals Leuven Leuven Belgium; ^4^ American OT Community; ^5^ Miami Neuroscience Institute Baptist Health South Florida Miami Florida USA

**Keywords:** clinical indicators, diagnosis, diagnostic delay, orthostatic tremor

## Abstract

**Background:**

The diagnosis of orthostatic tremor (OT) is challenging because of its non‐specific symptoms and a broad range of differential diagnosis, resulting in a complex and lengthy diagnostic journey.

**Objective:**

The aim was to assess the diagnostic delay in OT and to identify key clinical indicators as reported by patients.

**Methods:**

In 2019, the American OT community developed and distributed a web‐based survey titled “Symptoms and Diagnosis of Orthostatic Tremor (OT).” The survey included questions on demographics, the diagnostic process, and symptomatology.

**Results:**

An international cohort of 360 OT patients participated in the study, of which 147 with electromyography (EMG)‐confirmed and 213 symptom‐based OT. In the EMG‐confirmed OT group, respondents reported an average diagnostic delay of 7.4 (±8.5) years on average, attributed to delays at both the patient and physician level. A diagnosis was made by a movement disorders specialist in 75 (51.0%) of 147 cases. Misdiagnosis initially occurred in 57 (38.8%) of 147 patients, whereas 49 (33.3%) of 147 patients suspected OT before receiving a formal diagnosis. OT was commonly reported a progressive condition. The most frequent symptoms on standing that prompted patients to seek medical attention included: “shakiness/tremors in both legs” (92.5%), “feelings of unsteadiness or imbalance” (75.5%), “toe curling” (61.9%), and the “feeling of falling while standing” (45.6%). Notably, 70.0% of respondents reported experiencing anxiety at least occasionally. These findings closely align with the findings of the symptom‐based OT group.

**Conclusion:**

This study underscores a significant diagnostic delay in OT and identifies key clinical indicators, which could facilitate earlier diagnosis.

Orthostatic tremor (OT), also known as “shaky legs syndrome,” is a neurological disorder characterized by high‐frequency tremor (≥13 Hz) in the legs while standing, which resolves on sitting down.^1^ According to the consensus statement of the International Parkinson and Movement Disorder Society on Tremor, the diagnostic criteria for OT include: (1) a subjective sensation of unsteadiness while standing; (2) minimal clinical findings, often limited to fine, visible or occasionally palpable rippling of the leg muscles during stance; and (3) surface electromyography (EMG) demonstrating a tremor frequency of ≥13 Hz.^1^, ^2^ Although OT spans the entire age spectrum, it typically affects middle‐age or elderly individuals, predominantly women, and follows a progressive course.^3^, ^4^, ^5^, ^6^

OT is considered an orphan disorder, likely under‐recognized, with most of the available knowledge derived from case reports, small case series, and a few cohort studies—only four of which involved 40 or more patients.^5^, ^7^, ^8^, ^9^, ^10^ As a result, significant gaps in understanding remain, including the extent of the diagnostic delay. Although previous reports have noted an average delay of 7 or more years, with some patients waiting up to 44 years from symptom onset to diagnosis, a detailed analysis of the underlying causes of this delay is lacking.^5^, ^8^, ^9^, ^11^ One possible explanation for the diagnostic delay is the frequent lack of recognition of OT, largely because of its rarity and its presentation. Symptoms are felt non‐specific for those not aware of the disease characteristics. Moreover, standard neurological examination typically yields normal results, except for mild ataxia, partly because the tremor is often invisible and gait issues in the elderly are frequently multifactorial.^12^, ^13^ Consequently, reaching an accurate diagnosis often involves a lengthy and complex process including misdiagnosis, although the precise diagnostic journey remains unclear.^14^, ^15^, ^16^, ^17^

Identifying specific clinical indicators for OT is crucial for improving the recognition and reducing diagnostic delays. Several key indicators have been described in the literature, including the well‐known “helicopter sign”—a sound resembling helicopter blades heard during auscultation of leg muscles.^18^, ^19^ However, many physicians are unfamiliar with these characteristic features of OT and often do not perform specific examination techniques, such as holding a stethoscope on the leg, complicating the recognition and diagnosis of OT.^20^

In 2019, the American OT community conducted a patient‐initiated survey on OT symptoms and diagnosis, which was distributed globally. This effort resulted in an international OT cohort comprising 360 individuals from 24 countries. Patient involvement in research enhances its relevance by addressing specific needs and concerns, ensuring that findings are more targeted and beneficial to the OT community. Moreover, international funding agencies are increasingly advocating for patient engagement, highlighting its importance in producing meaningful and impactful healthcare research.^21^

This study aims to address the issue of diagnostic delay in OT and identify clinical indicators based on patient reported experiences through a comprehensive cohort analysis. By highlighting these clinical indicators, the study will provide a foundation for an improved diagnostic framework, enabling physicians to use specific testing methods for OT recognition and ultimately reducing diagnostic delays. The study will assess: (1) the demographic characteristics of the patient cohort; (2) the extent of diagnostic delays and the physicians involved in the diagnostic process; (3) the most commonly reported symptoms and complaints; and (4) associated comorbidities.

## Methods

### Participants

We included all respondents who: (1) completed the “Symptoms and Diagnosis of Orthostatic Tremor (OT)” survey described below; and (2) had a diagnosis of OT. Respondents were recruited through various international OT support groups active on social media. The included respondents were divided into two groups: those with EMG confirmation of OT standing (EMG‐confirmed OT), and those without EMG confirmation and those who or were uncertain about having undergone one (symptom‐based OT). Data was collected anonymously, therefore, written informed consent was not required.

### Survey

In 2019, the American OT community developed and launched a web‐based survey titled “Symptoms and Diagnosis of Orthostatic Tremor (OT)” on orthostatictremor.org. Survey data was collected in May 2019 using the online platform SurveyMonkey.

Respondents could easily and independently access the survey through the OT community website, either by searching online or via referrals from various international OT support groups. The survey was available in English and, following translations by native speakers with above average proficiency in English, was also offered in French, Spanish, and Dutch.

The survey comprised 57 questions, primarily in a multiple‐choice format with predefined answer options. Additionally, open fields were provided for respondents to offer alternative answers where applicable (see File S1).

### Analyses

The following outcomes, along with the corresponding survey items, were selected for analysis:

(1) Demographic characteristics: age, gender, country of residence, and ethnicity.

(2) Diagnostic delay and physicians involved: age at symptom onset, age at seeking medical help, age at diagnosis, medical professionals consulted, the number and type of first medical professional visited, initial diagnoses, diagnoses received before OT, the practitioner who diagnosed OT, and whether patients suspected OT before diagnosis.

(3) Symptoms and complaints: occurrence, severity, and progression before and after OT‐diagnosis, which are categorized into five groups: symptoms on standing, alleviating factors, exacerbating factor, symptoms in the arms, and other symptoms.

(4) Neurological and mental comorbidities: additional neurological diagnoses and other comorbidities.

Results for respondents with EMG‐confirmed OT are presented in the main text and tables, while findings from those with symptom‐based OT are summarized in Appendix S1. Descriptive data from both groups are given in percentages (n = x) or in mean (range). Missing values were not replaced.

## Results

### Participants

A total of 360 respondents were included in the study. Of these, 147 reported having their diagnosis confirmed by EMG recordings during standing (EMG‐confirmed OT group) with results provided below. Results for the respondents in the symptom‐based OT (n = 213) group are provided in the Appendix S1, and Appendix Tables S1–S3.

### Demographic Characteristics

In the “EMG‐confirmed OT” group, 81.6% of respondents were female (n = 120). The mean age was 65.5 years (range: 39–90). The majority of respondents resided in the United States (32.7%, n = 48), the United Kingdom (15.0%; n = 22), and the Netherlands (14.3%, n = 21), while 96.6% were of Caucasian origin (n = 142). Detailed information is presented in Table 1 and Figure S1.

**TABLE 1:** Patient demographics

Characteristics	Confirmed EMG (n = 147)
Gender (n)	
Male	27 (18.4%)
Female	120 (81.6%)
Age (y, range)	65.5 (39–90)
Ethnicity (n)	
Caucasian	142 (96.6%)
Hispanic	3 (2%)
Black	1 (0.7%)
Asian	1 (0.7%)
Country of residence (n)	
United States	48 (32.7%)
United Kingdom	22 (15%)
The Netherlands	21 (14.3%)
France	20 (13.6%)
Australia	12 (8.2%)
Canada	12 (8.2%)
Other^a^	12 (8.2%)

*Note*: Values are given either given in absolute numbers (percentage of total), unless otherwise indicated.

^a^ Belgium, Brazil, Italy, Luxembourg, Mexico, New Zealand, Sweden, Switzerland.

Abbreviation: EMG, electromyography.

### Diagnostic Delay and Involved Physicians

The mean age of OT onset was 50.3 years (range: 8–70). On average, respondents waited 3.4 years (range: 1–27) after symptom onset to seek medical help (patient delay), followed by an additional 4.3 years (range: 1–50) to receive an OT diagnosis (doctor delay), resulting in a total diagnostic delay of 7.4 years (range: 0–56) (Table 2).

**TABLE 2:** Diagnostic characteristics

Characteristics		Confirmed EMG (n = 147)
Age of onset (y)		50.3 (8–70)
Age at diagnosis (y)		57.7 (30–74)
Diagnostic delay (y)	Total	7.4 (0–56)
	No. of years before discussing with physician	3.4 (1–27)
	No. of years to receive OT diagnosis after first speaking with physician	4.3 (1–50)
No. of physicians seen before receiving diagnosis (n)		3.2 (1–8+)
Practitioner symptoms first discussed with (n)	General practitioner	93 (63.3%)
	General neurologist	32 (21.8%)
	Movement disorders neurologist	7 (4.8%)
	Orthopedics	3 (2.0%)
	Psychiatrist/psychologist	4 (2.7%)
	Other^a^	8 (5.4%)
Diagnoses received before OT^b^ (n)	Orthostatic tremor on first visit	20 (13.6%)
	No diagnosis on first visit	64 (43.5%)
	“Nothing is wrong”	6 (4.1%)
	Essential tremor	12 (8.2%)
	Mental/psychological	25 (17%)
	Anxiety	22 (15%)
	PD	8 (5.4%)
	Spinal disorder	2 (1.4%)
	RLS	7 (4.7%)
Practitioner that diagnosed OT (n)	General practitioner	2 (1.4%)
	General neurologist	69 (46.9%)
	Movement disorders neurologist	75 (51%)
	Other^c^	1 (0.7%)

*Note*: Values are given either given in average number (range) or in absolute numbers (percentage of total).

^a^ Cardiologist, chiropractor, otorhinolaryngologist, nurse practitioner, physical therapist, rheumatologist, transplant doctor.

^b^ Multiple patients received multiple diagnoses before their diagnosis of OT. Percentages are calculated based on total patient number (n = 147).

^c^ Psychiatrist/psychologist.

Abbreviations: EMG, electromyography; OT, orthostatic tremor; PD, Parkinson's disease; RLS, restless leg syndrome.

The definitive diagnosis of OT was most commonly made by a neurologist, including general neurologist (46.9%, n = 69) and movement disorder neurologist (51.0%, n = 75) (Table 2). Notably, a subset of respondents (33.3%, n = 49) suspected OT themselves before receiving a formal diagnosis.

Before receiving their OT diagnosis, respondents consulted an average of three medical professionals (range: 1–8+), with seven respondents (4.1%) seeing more than eight professionals. The majority of respondents' first consultations were with a general practitioner (primary care physician) (63.3%, n = 93), a general neurologist (21.8%, n = 32), or a movement disorder neurologist (4.8%, n = 7).

Among respondents, 13.6% (n = 20) were diagnosed with OT at their first consultation, whereas 47.6% (n = 70) received no diagnosis. Additionally 25.2% (n = 37) were initially misdiagnosed with one or more other conditions, including essential tremor (ET) (n = 12), Parkinson's disease (PD) (n = 8), anxiety (n = 22), or other psychological disorders (n = 25) (Table 2). On average, respondents experienced one misdiagnosis (range: 0–7) before receiving a formal OT diagnosis.

### Symptoms and Complaints

**FIG. 1:**
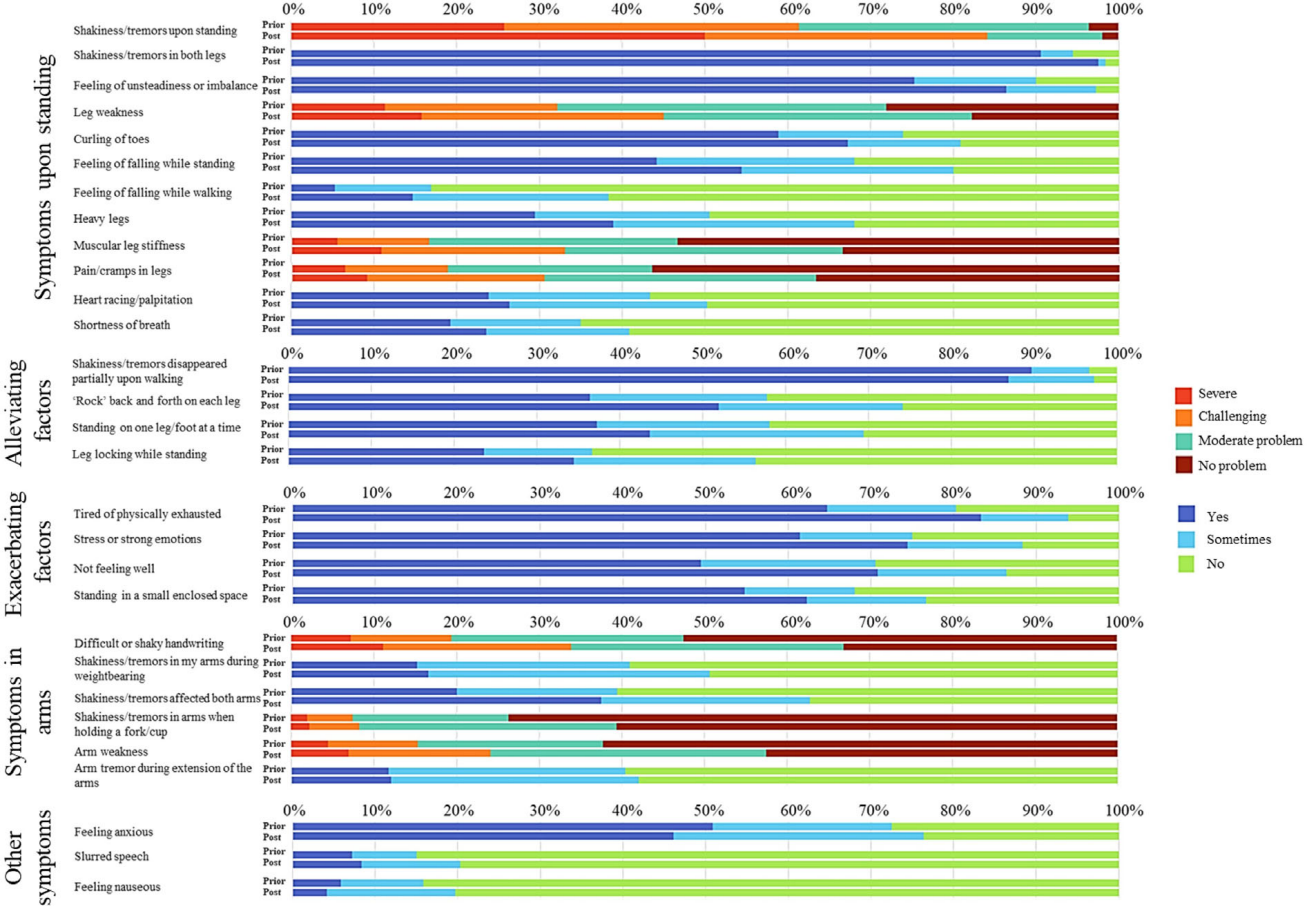
Symptoms before and post orthostatic tremor (OT) diagnosis. Symptoms were categorized into five distinct categories. The percentage of respondents experiencing each symptom, along with the severity of several symptoms both before and after receiving an OT diagnosis, is presented.

The most commonly reported symptom prompting respondents to seek medical help was “shakiness/tremors in both legs upon standing” (92.5%, n = 136), with 59.9% (n = 88) describing this as challenging or severe (Fig. 1). Other frequently reported symptoms included a “feeling of unsteadiness or imbalance” (75.5%, n = 111), “curling of the toes” (61.9%, n = 91), and a “feeling of falling while standing” (45.6%, n = 67).

Walking was the most common strategy to alleviate tremors, providing relief for 88.4% (n = 130) of the respondents. The most significant exacerbating factors were “tiredness or physical exhaustion” (64.5%, n = 95) and “stress (or other strong emotions)” (63.9%, n = 94), followed by “not feeling well” (51.0%, n = 75), and “standing in a small enclosed space” (59.2%, n = 87).

In addition to symptoms in the legs, 21.1% (n = 31) of respondents reported symptoms in the arms, and 70.0% (n = 103) reported feeling anxious at least occasionally. Notably, when including the respondents who experienced symptoms only occasionally, the overall prevalence of these symptoms and factors, including those of most common reported symptoms, alleviating and exacerbating factors, and additional symptoms, are even higher (Fig. 1).

Symptoms were generally described as progressive, with both the number and severity of reported symptoms increasing after diagnosis. Before diagnosis, respondents reported a mean of 15 symptoms (range: 2–27), compared to 19 symptoms (range: 1–28) after diagnosis. Additionally, after diagnosis the reported symptoms and factors tend to be reported by more respondents compared to before diagnosis, aside of the alleviating factor “walking” that seemed to be reported by fewer respondents after diagnosis (Appendix Figs. S2 and S3).

### Neurological and Mental Comorbidities

Most respondents (62.6%, n = 92) reported having no neurological or mental comorbidities. The remaining 37.4% (n = 55) indicated the presence of anxiety (19.7%, n = 29), depression (15%, n = 22), ET (8.2%, n = 12), restless leg syndrome (5.4%, n = 8), or other comorbidities (Table 3).

**TABLE 3:** Comorbidities

Disorder	Confirmed EMG (n = 147)
Orthostatic tremor only	92 (62.6%)
Anxiety	29 (19.7%)
Depression	22 (15%)
Essential tremor	12 (8.2%)
Restless legs syndrome	8 (5.4%)
Ataxia	2 (1.4%)
Dystonia	4 (2.7%)
Parkinson's disease/parkinsonism	4 (2.7%)
Orthostatic myoclonus	3 (2%)
Epilepsy	2 (1.4%)
Fibromyalgia	1 (0.7%)
Dementia	1 (0.7%)
Myasthenia gravis	1 (0.7%)

*Note*: Values are given either given in absolute numbers (percentage of total).

Abbreviation: EMG, electromyography.

### EMG‐Confirmed Versus Symptom‐Based OT

Most findings from the EMG‐confirmed OT group closely aligned with those from the symptom‐based OT group (see Appendix S1), with only minor differences in factors such as country of residence, time to seek medical help, diagnosis received before OT, awareness of OT before diagnosis, the reported symptom “muscular leg stiffness,” and the number of reported symptoms.

## Discussion

Our analysis of one of the largest OT cohorts, as indicated by the findings from the EMG‐confirmed OT group, suggests that patients with OT experience a significant diagnostic delay because of both patient and doctor related factors. OT is often not immediately recognized, as evidenced by the high number of medical professionals consulted and the frequent misdiagnosis before receiving a formal diagnosis. Half of the patients were ultimately diagnosed by a neurologist specializing in movement disorders. A subset of respondents had already suspected they might have OT before receiving the formal diagnosis. At the time of their first consultation, most respondents did not receive a diagnosis. Among those who did, many were initially misdiagnosed with anxiety. The most commonly reported symptoms prompting respondents to seek medical help included shakiness/tremors in both legs on standing, a feeling of unsteadiness or imbalance, toe curling, and the sensation of falling. Anxiety was also frequently reported, both as a symptom and as a comorbidity. After diagnosis, both the number and severity of symptoms tended to increase during follow‐up. Notably, the findings from the EMG‐confirmed OT group closely aligned with those from the symptom‐based OT group.

Although our findings from both the EMG‐confirmed and symptom‐based OT group align with previous reports of an average diagnostic delay of ≥7 years, this study is the first to specifically differentiate between patient delay and doctor delay.^5^, ^11^ In PD, for example, the average diagnostic delay in diagnosis is approximately 3.3 years, with some studies reporting even shorter delays, underscoring the notably prolonged delay in OT compared to other movement disorders.^14^, ^22^, ^23^ Patient delay, although common, remains challenging to address, because it relies heavily on public awareness and the availability of reliable information online to guide patients toward the appropriate subspecialty. Therefore, reducing the overall diagnostic delay depends primarily on addressing the prolonged doctor delay, which likely stems from a general lack of awareness about rare disorders like OT and the nonspecific nature of its symptoms.^13^ This is further supported by the considerable number of physicians consulted before patients receive a formal OT diagnosis. Many physicians likely fail to recognize OT symptoms, leading to misdiagnosis and patient referrals to specialists other than movement disorders neurologists. Long wait times for subspecialty providers further exacerbate these delays, emphasizing the need for timely referrals to the appropriate specialists. Additionally, in the absence of clear neurological signs, some patients may be diagnosed based solely on their anxiety symptoms. Given the high prevalence of anxiety in OT patients, as observed in our study and consistent with previous reports, as well as the often positive response to clonazepam, likely explains why anxiety remains the most frequent misdiagnosis.^15^, ^24^ Increasing awareness of OT among both physicians and patients is crucial to reduce diagnostic delays.

Consistent with the clinical diagnostic criteria for OT and existing literature, our study identified the “feeling of unsteadiness or imbalance” as an important indicator of OT.^1^, ^2^, ^5^, ^7^, ^8^, ^9^ However, many of the symptoms reported by respondents, including the most commonly noted “shakiness/tremors in both legs upon standing,” are not part of the official diagnostic criteria. It is often suggested that patients may not directly perceive the tremor itself, but instead experience its secondary effects, such as imbalance. Whether respondents reported “shakiness/tremors in both legs upon standing” because of retrospective awareness following diagnosis or as direct symptoms remains unclear. Nevertheless, this symptom was reported by the majority of respondents and has also been sporadically mentioned as a clinical indicator of OT.^16^, ^20^, ^25^ Other notable indicators, such as fear of falling, which may correspond to the “feeling of falling while standing” reported in our study, and “curling of the toes,” have also been referenced in prior research, although less frequently.^6^, ^7^, ^16^, ^17^, ^18^, ^20^, ^25^ Additionally, some respondents reported experiencing tremor in their upper limbs, these symptoms are less prevalent compared to those occurring while standing.^7^, ^8^, ^26^ The exact nature of upper limb tremors in OT remains unclear. Some studies suggests that OT may be a variant of ET because of overlapping symptoms,^25^, ^26^ whereas other research propose that postural arm tremor could either be a feature of coexisting ET or an intrinsic part of OT itself.^8^, ^9^, ^18^, ^26^

When comparing findings from both the EMG‐confirmed and symptom‐based OT groups to existing literature, our study observed a predominance of females and an onset of OT in middle‐age and elderly individuals. These results align with the four large OT cohorts previously reported.^5^, ^7^, ^8^, ^9^ However, our cohort showed a higher percentage of female participants compared to earlier studies. Ethnicity has not been thoroughly assessed in OT research to date. In our study, the majority of respondents were of Caucasian descent. Whether this reflects a selection bias because of the survey distribution or indicates a true demographic trend remains unclear. It is worth noting that Parkinson's disease (PD) has also been found to be more prevalent in individuals of Caucasian descent.^27^ Overall, the demographic characteristics of our study population are consistent with existing literature, supporting the external validity of our findings.

This study has several limitations. First, the survey was developed by the American OT community and subsequently presented to the investigators. Although some questions may not have been fully clear or comprehensive from a research perspective, the survey reflects concerns prioritized by the patient community. Furthermore, consultation with the survey creators, who are also co‐authors, ensured alignment with the study's objectives. With the growing emphasis on patient involvement in research, this patient‐led initiative provides unique insights that might not have emerged through traditional investigator‐led studies.^21^ Second, OT diagnoses were self‐reported, which could introduce misreporting. However, this risk was mitigated by distributing the survey through the OT community website, with recruitment primarily facilitated by various OT support groups. Additionally, the large number of respondents, the consistent findings between the EMG‐confirmed and symptom‐based OT groups, and the alignment of demographic characteristics with expectations from the literature lend credibility to the results. Third, the retrospective nature of data collection may have introduced recall bias. Finally, the anonymous nature of the survey limited the availability of detailed respondent information, restricting the ability to conduct more nuanced analyses or draw deeper correlations between outcomes.

The delay in OT diagnosis arises from both patient hesitation in seeking medical help and the prolonged diagnostic process, which often involves consulting multiple physicians and encountering frequent misdiagnoses. This is likely because of the general unfamiliarity with OT among healthcare providers. Consistent with existing literature, a significant proportion of respondents reported symptoms such as a feeling of unsteadiness or imbalance and anxiety. However, symptoms less frequently discussed in the literature but commonly reported by our respondents, both with EMG‐diagnosed and symptom‐based OT, included shakiness or tremors in both legs on standing, curling of the toes, and the sensation of falling. Recognizing these patient‐reported indicators could help physicians consider OT earlier when patients present with these symptoms.

## Author Roles

(1) Research project: A. Conception, B. Organization, C. Execution; (2) Statistical Analysis: A. Design, B. Execution, C. Review and Critique; (3) Manuscript: A. Writing of the First Draft, B. Review and Critique.

W.B.: 1A, 1B, 1C, 2A, 2B, 3A

K.W.: 1A, 1B, 2A, 1B

B.S.: 1A, 3B

S.D.: 1A, 3B

M.M.: 1A, 1B, 3B

R.B.: 3B

D.T.: 1A, 3B

A.R.: 1A, 2C, 3B

## Disclosures

**Ethical Compliance Statement:** We confirm that we have read the Journal's position on issues involved in ethical publication and affirm that this work is consistent with those guidelines. Written informed consent was obtained for the data outside of standard patient care. A waiver of informed consent was obtained from the local medical ethical committee for the data that is part of standard patient care.

**Funding Sources and Conflict of Interests:** All authors report no declarations of interest. This research did not receive any specific grant from funding agencies in the public, commercial, or not‐for‐profit sectors.

**Financial Disclosure for the Previous 12 Months:** B.S., S.D., M.M., and D.R.T. have no financial disclosures for the previous 12 months to report. W.A.B. received a grant from Stichting Het Remmert Adriaan Laan Fonds paid to the institution. The funding source had no role in the study design; in the collection, analysis, and interpretation of data; in the writing of the report; and in the decision to submit the article for publication. R.M.A.d.B. has not received any funding related to the current research. R.M.A.d.B. received research grants from Medtronic, Bial, ZonMw, AMC Foundation, ROMO Foundation, and Stichting ParkinsonFonds, all paid to the institution. A.F.v.R. has not received any funding related to the current research. A.F.v.R. received a research grant from Stichting De Merel, paid to the institution.

## Supporting information


**Figure S1.** Overview respondents by country of residence. Number of respondents categorized by country for both EMG‐confirmed OT and symptom‐based OT (A), for EMG‐confirmed OT only (B) and symptom‐based OT only (C).

**Figure S2.** Symptoms upon standing prior to and post OT diagnosis. The percentage of respondents experiencing each symptom upon standing, along with the severity of several symptoms both before and after receiving an OT diagnosis, are presented for both EMG‐confirmed OT and symptom‐based OT.

**Figure S3.** Symptoms prior to and post OT diagnosis. The percentage of respondents experiencing each symptom, as well as the severity of several symptoms before and after an OT diagnosis, is presented for both EMG‐confirmed OT and symptom‐based OT across the categories of ‘other symptoms,’ ‘symptoms in arms,’ ‘alleviating factors,’ and ‘exacerbating factors.’

**Appendix S1.** Includes the summarized result from the symptom‐based OT patients.

**File S1.** Includes the web‐based survey titled ‘Symptoms and Diagnosis of Orthostatic Tremor (OT)’ used to collect data.

**Table S1.** Patient Demographics.

**Table S2.** Diagnostic Characteristics.

**Table S3.** Comorbidities.

## Data Availability

The data that support the findings of this study are available from the corresponding author upon reasonable request.
