# Supplementary figures and images for: Identifying the Diagnostic Challenges and Indicators of Orthostatic Tremor: Patient Perspectives
Source: Mov Disord Clin Pract. 2025 Apr 23;12(8):1124–31. doi: 10.1002/mdc3.70081 (PMC12371454; doi:10.1002/mdc3.70081)

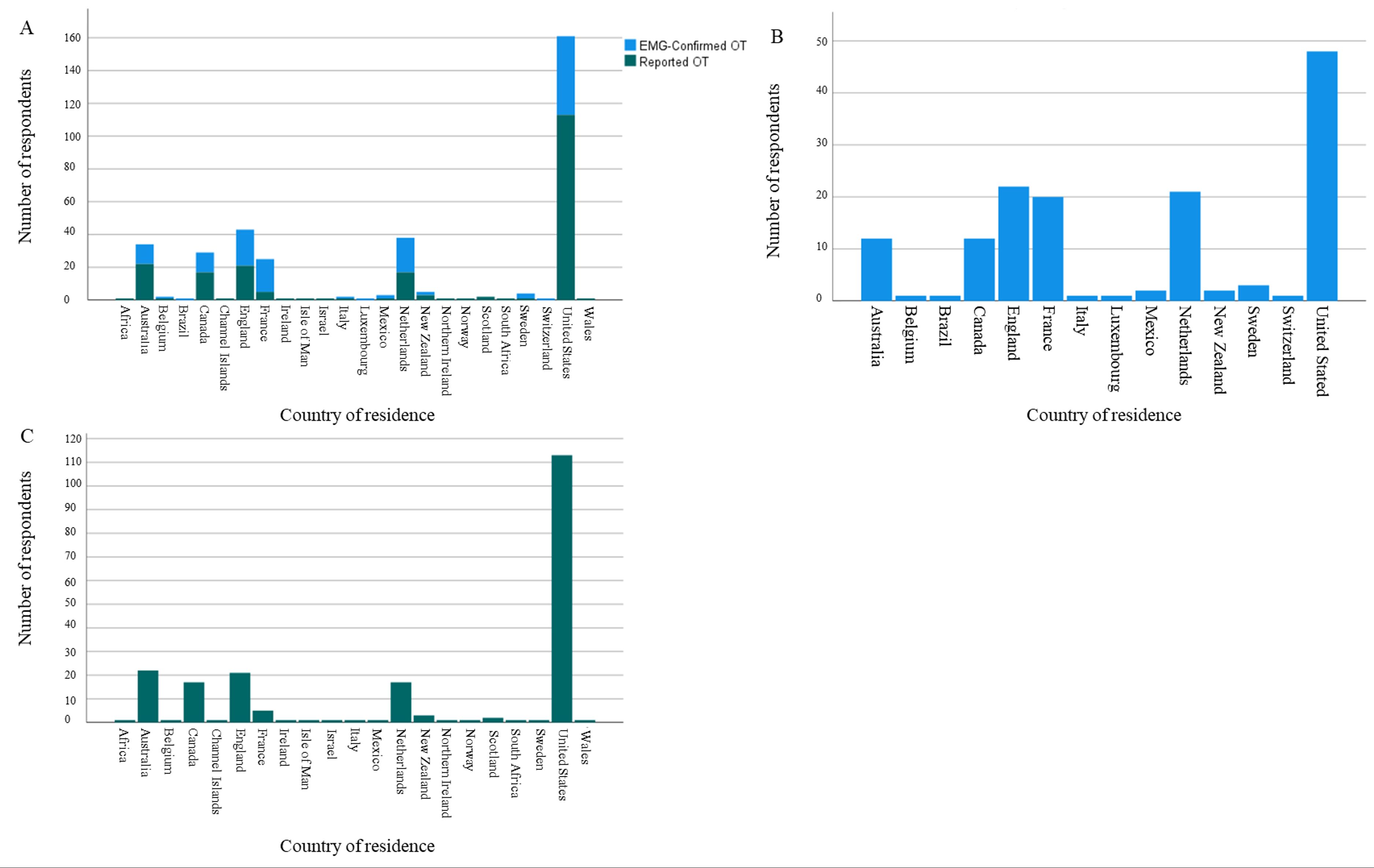

Supplement: Supplementary file 1 — Figure S1. Overview respondents by country of residence. Number of respondents categorized by country for both EMG‐confirmed OT and symptom‐based OT (A), for EMG‐confirmed OT only (B) and symptom‐based OT only (C). [file MDC3-12-1124-s006.jpg]

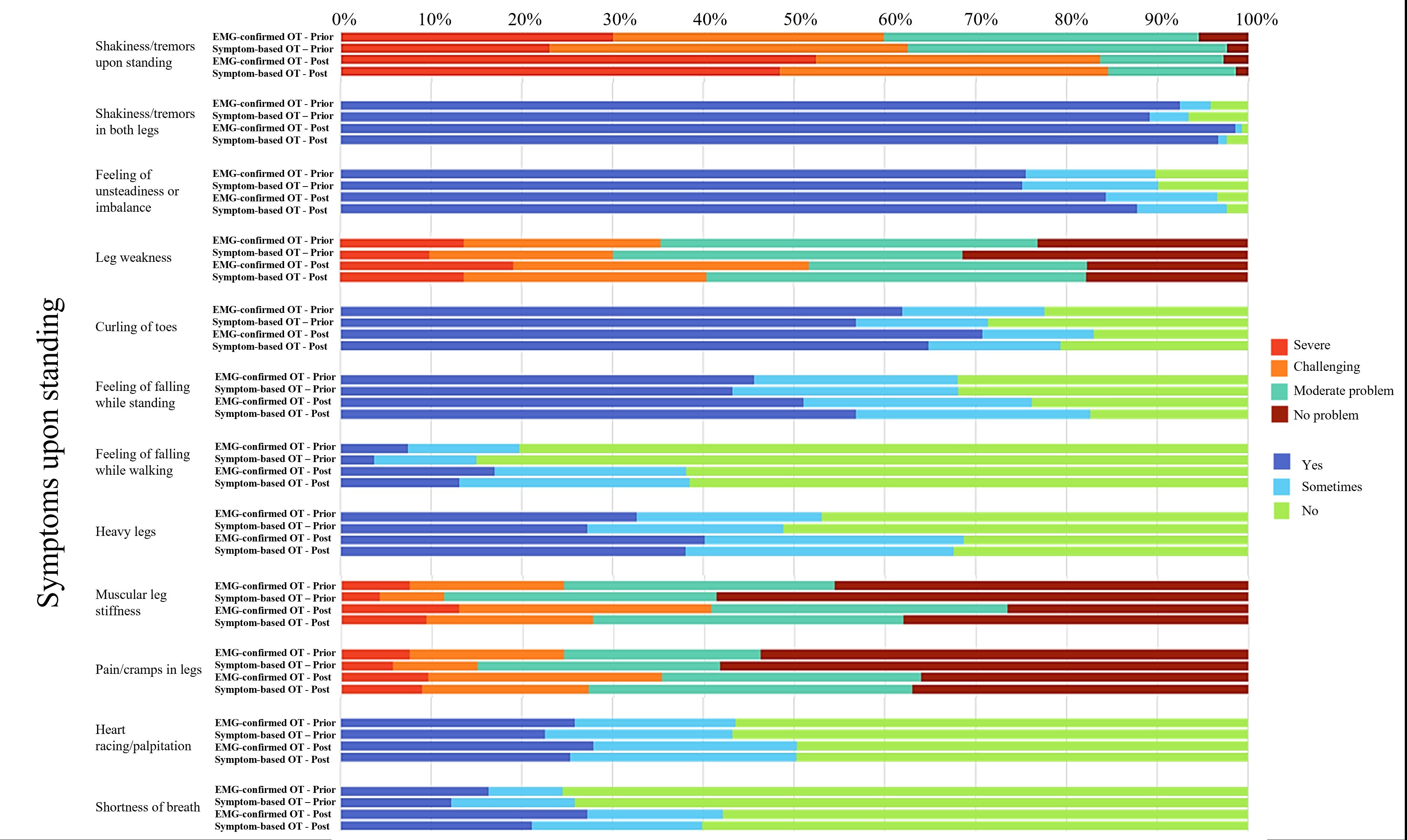

Supplement: Supplementary file 2 — Figure S2. Symptoms upon standing prior to and post OT diagnosis. The percentage of respondents experiencing each symptom upon standing, along with the severity of several symptoms both before and after receiving an OT diagnosis, are presented for both EMG‐confirmed OT and symptom‐based OT. [file MDC3-12-1124-s008.jpg]

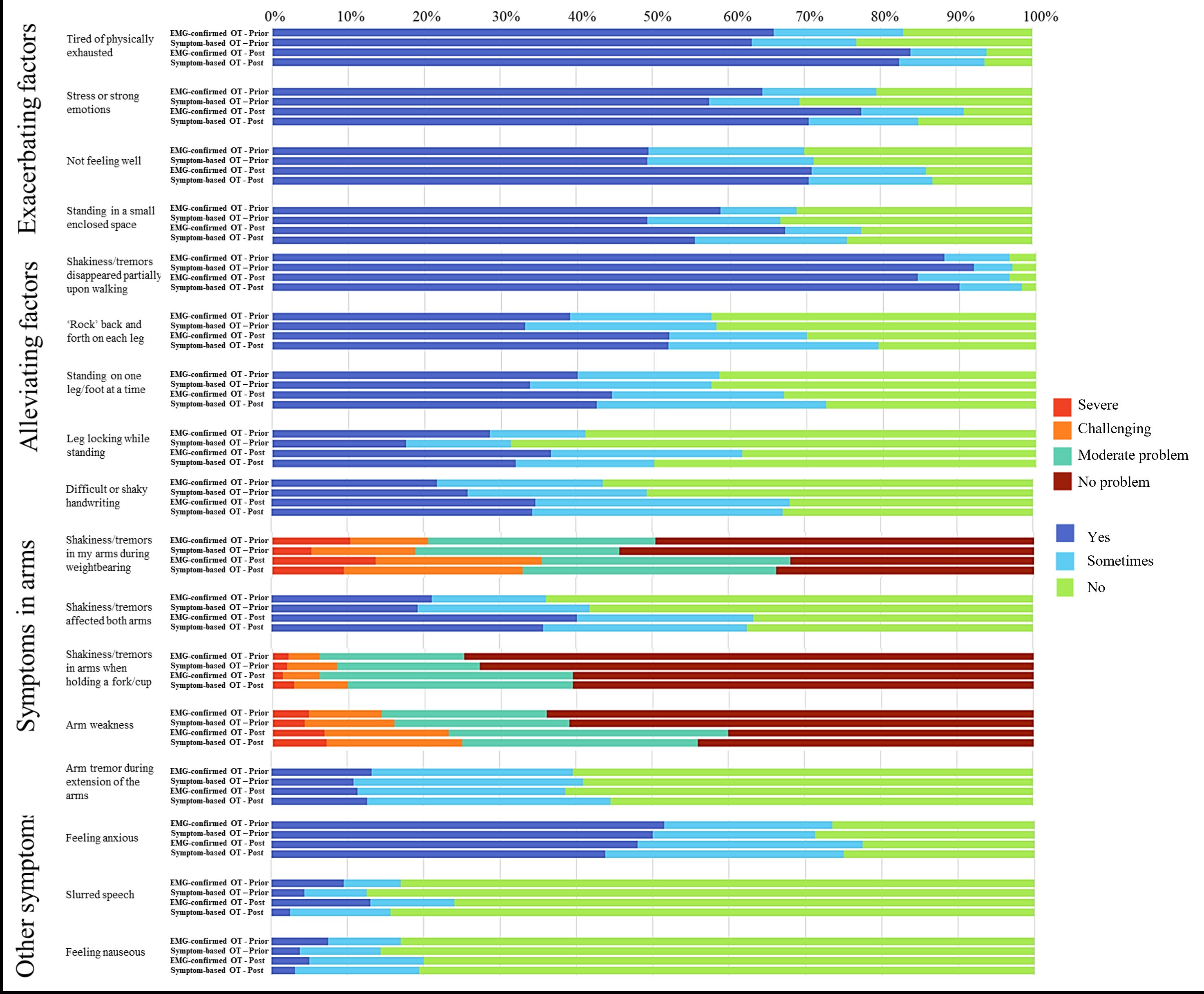

Supplement: Supplementary file 3 — Figure S3. Symptoms prior to and post OT diagnosis. The percentage of respondents experiencing each symptom, as well as the severity of several symptoms before and after an OT diagnosis, is presented for both EMG‐confirmed OT and symptom‐based OT across the categories of ‘other symptoms,’ ‘symptoms in arms,’ ‘alleviating factors,’ and ‘exacerbating factors.’ [file MDC3-12-1124-s004.jpg]
