# Supplementary material for: Identifying the Diagnostic Challenges and Indicators of Orthostatic Tremor: Patient Perspectives
Source: Mov Disord Clin Pract. 2025 Apr 23;12(8):1124–31. doi: 10.1002/mdc3.70081 (PMC12371454; doi:10.1002/mdc3.70081)
Supplement: Supplementary file 4 — Appendix S1. Includes the summarized result from the symptom‐based OT patients. [file MDC3-12-1124-s003.docx]

Identifying the Diagnostic Challenges and Indicators of Orthostatic Tremor: Patient Perspectives

# Supplementary Appendix

**Results**

*Demographic characteristics*

In the ‘symptoms-based OT’ group, 78.4% of respondents were female (n=167). The mean age was 67.1 years (range: 33-86). The majority of respondents resided in the United States (53.1%, n=113), the United Kingdom (10.3%; n=22), and Australia (10.3%, n=22), while 97.7% were of Caucasian origin (n=208). Detailed information is displayed in Table S1 and Figure S1.

*Diagnostic delay and involved physicians*

The mean age of OT onset was 52.1 years (range: 16-80). On average, respondents waited 4.1 years (range: 0-40) after symptom onset to seek medical help (patient delay), followed by an additional 4.0 years (range: 0-38) to receive an OT diagnosis (doctor delay), resulting in a total diagnostic delay of 7.8 years (range: 0-42) on average (Table S2).

The definitive diagnosis of OT was most commonly made by a neurologist, including general neurologist (54.5%, n=116) and movement disorder neurologist (43.2%, n=92) (Table S2). Notably, a subset of the respondents (43.2%, n=92) suspected OT themselves prior to receiving a formal diagnosis.

Before receiving their OT diagnosis, respondents consulted an average of three medical professionals (range: 1-8+), with four respondents (1.9%) seeing more than eight medical professionals. The majority of respondents’ first consultations were with a general practitioner (primary care physician) (67.6%, n=144), a general neurologist (19.7%, n=42) or a movement disorder neurologist (8.9%, n=19).

Among respondents, 20.7% (n=32) were diagnosed with OT at their first consultation, while 24.5% (n=52) received no diagnosis. Additionally, 38% (n=81) were initially misdiagnosed with one or more other conditions, including essential tremor (ET, n=24), Parkinson’s disease (PD, n=9), anxiety (n=20), or other psychological disorders (n=14) (Table S2). On average, respondents experienced one misdiagnosis (range: 0–6) before receiving a formal OT diagnosis.

*Symptoms and complaints*

The most commonly reported symptom prompting respondents to seek medical help was ‘shakiness/tremors in both legs upon standing’ (89.2%, n=190), with 62.4% (n=133) describing this as challenging or severe (Supplementary Figure 2). Other frequently reported symptoms included a ‘feeling of unsteadiness or imbalance’ (75.1%, n=160), ‘curling of the toes’ (56.8%, n=121), and the ‘feeling of falling while standing’ (43.2%, n=92).

Walking was the most common strategy to alleviate tremors, providing relief for 90.6% (n=193) of the respondents. The most significant exacerbating factors were ‘tiredness or physical exhaustion’ (64.8%, n=138) and ‘stress (or other strong emotions)’ (59.6%, n=127), followed by ‘standing in a small enclosed space’ (51.5%, n=110), and ‘not feeling well’ (48.4%, n=103).

In addition to symptoms in the legs, 19.2% (n=41) of respondents reported symptoms in the arms, and 74.2% (n=158) reported feeling anxious at least occasionally. Notably, when including the respondents who experienced symptoms only occasionally, the overall prevalence of these symptoms and factors, including those of most common reported symptoms, alleviating and exacerbating factors, and additional symptoms, are even higher (Figure S2).

Symptoms were generally described as progressive, with both the number and severity of reported symptoms increasing after diagnosis. Before diagnosis, respondents reported a mean of 10 symptoms (range: 0-22), compared to 13 symptoms (range: 1-23) after diagnosis. Additionally, after diagnosis the reported symptoms and factors tend to be reported by more respondents compared to before diagnosis, aside of the alleviating factor ‘walking’ that seemed to be reported by fewer respondents after diagnosis (Figure S2 and S3).

*Neurological and mental comorbidities*

Most respondent (56.8%, n=121) reported having no neurological or mental comorbidities. The remaining 37.4% (n=55) indicated the presence of anxiety (23.9%, n=51), depression (16.9%, n=36), ET (14.6%, n=31), restless leg syndrome (4.2%, n=9), or other comorbidities (Table S3).

**Legend files**

Supplementary Appendix Figure S1

Title:

Overview of the number of respondents by country of residence.

Text:

A) The total number of respondents (i.e., EMG-confirmed OT and symptom-based OT) per country, B) the number of EMG-confirmed OT respondents per country, and C) the number of symptom-based OT respondents per country.

Supplementary Appendix Figure S2

Title:

Symptoms upon standing prior to and post OT diagnosis.

Text:

The percentage of respondents (i.e., EMG-confirmed OT and symptom-based OT) experiencing symptoms upon standing, along with the severity of several symptoms both before and after receiving an OT diagnosis, are presented.

Supplementary Appendix Figure S3

Title:

Symptoms prior to and post OT diagnosis.

Text:

Symptoms of four distinct categories are given for both the confirmed OT and symptom-based OT group. The percentage of respondents experiencing each symptom, along with the severity of several symptoms both before and after receiving an OT diagnosis, are presented.

**Tables**

Table S1. *Patient Demographics*.

Values are given either given in absolute numbers (percentage of total), unless otherwise indicated.

Abbreviations: n= number of subjects, y= years

*Africa, Belgium, Brazil, Channel Islands, Ireland, Isle of Man, Israel, Italy, Luxembourg, Mexico, New Zealand, Northern Ireland, Norway, Scotland, South Africa, Sweden, Switzerland, Wales.

Abbreviations: EMG= electromyography, n.k.= not known

a= Duration of the disease from diagnosis till inclusion in the study

b= Data recorded from the m. tibialis anterior left and/or m. tibialis anterior right

*= Switched the DBS off

| Characteristics | | Total (n=360) | Confirmed EMG (n=147) | Reported EMG (n=213) |
| --- | --- | --- | --- | --- |
| Gender (n) | Male | 73 (20.3%) | 27 (18.4%) | 46 (21.6) |
|  | Female | 287 (79.7%) | 120 (81.6%) | 167 (78.4) |
| Age (y, range) | | 66.4 (range 33-90) | 65.5 (39-90) | 67.1 (33-86) |
| Ethnicity (n) | Caucasian | 350 (97.1%) | 142 (96.6%) | 207 |
|  | Hispanic | 6 (1.7%) | 3 (2%) | 3 |
|  | Black | 2 (0.6%) | 1 (0.7%) | 1 |
|  | Asian | 2 (0.6%) | 1 (0.7%) | 1 |
| Country of Residence (n) | United States | 161 (44.7%) | 48 (32.7%) | 113 |
|  | United Kingdom | 43 (11.9%) | 22 (15%) | 22 |
|  | The Netherlands | 38 (10.6%) | 21 (14.3%) | 21 |
|  | Australia | 34 (9.4%) | 12 (8.2%) | 17 |
|  | Canada | 29 (8.1%) | 12 (8.2%) | 17 |
|  | France | 25 (6.9%) | 20 (13.6%) | 5 |
|  | Others* | 30 (8.3%) | 12 (8.2%) | 18 |

Table S2. *Diagnostic Characteristics*.

Abbreviations: EMG= electromyography, n.k.= not known

a= Duration of the disease from diagnosis till inclusion in the study

b= Data recorded from the m. tibialis anterior left and/or m. tibialis anterior right

*= Switched the DBS off

| Characteristics | | Total (n=360) | Confirmed EMG (n=147) | Reported EMG (n=213) |
| --- | --- | --- | --- | --- |
| Age of onset (y) | | 51.3 (8-80) | 50.3 (8-70) | 52.1 (16-80) |
| Age at diagnosis (y) | | 59 | 57.7 (30-74) | 59.9 (28-82) |
| Diagnostic delay (y) | Total |  |  |  |
|  | # of years before discussing with physician | 3.9 (1-40) | 3.4 (1-27) | 4.1 (0-40) |
|  | # of years to receive OT diagnosis after first speaking with physician | 4.1 ( 0-50) | 4.3 (1-50) | 4.0 (0-38) |
| # of physicians seen before receiving diagnosis (n) | | 3 (1-8+) | 3.2 (1-8+) | 2.9 (1-8+) |
| Practitioner symptoms first discussed with (n) | General Practitioner | 237 (65.8%) | 93 (63.3%) | 144 (67.6%) |
|  | General Neurologist | 74 (20.6%) | 32 (21.8%) | 42 (19.7%) |
|  | Movement Disorders Neurologist | 26 (7.2%) | 7 (4.8%) | 19 (8.9%) |
|  | Orthopedics | 6 (1.7%) | 3 (2.0%) | 3 (1.4%) |
|  | Psychiatrist/ psychologist | 5 (1.4%) | 4 (2.7%) | 1 (0.5%) |
|  | Other* | 12 (3.3%) | 8 (5.4%) | 4 (1.9%) |
| Diagnoses received prior to OT | Orthostatic tremor on first visit | 52 (14.4%) | 20 (13.6%) | 32 (15%) |
|  | No diagnosis on 1^st^ visit | 108 (30%) | 64 (43.5%) | 44 (20.7%) |
|  | “Nothing is wrong” | 14 (3.9%) | 6 (4.1%) | 8 (3.8%) |
|  | Essential tremor | 36 (10%) | 12 (8.2%) | 24 (11.2%) |
|  | Mental/psychological | 39 (10.8%) | 25 (17%) | 14 (6.6%) |
|  | Anxiety | 42 (11.6%) | 22 (15%) | 20 (9.4%) |
|  | PD | 17 (4.7%) | 8 (5.4%) | 9 (4.2%) |
|  | Spinal disorder | 8 (2.2%) | 2 (1.4%) | 6 (2.8%) |
|  | RLS | 10 (2.7%) | 7 (4.7%) | 3 (1.4%) |
| Practitioner that diagnosed OT (n) | General Practitioner | 5 (1.4%) | 2 (1.4%) | 3 (1.4%) |
|  | General Neurologist | 185 (51.4) | 69 (46.9%) | 116 (54.5%) |
|  | Movement Disorders Neurologist | 167 (46.4%) | 75 (51%) | 92 (43.2%) |
|  | Other** | 3 (0.8%) | 1 (0.7%) | 2 (0.9%) |

Values are given either given in average number (range) or in absolute numbers (percentage of total).

Abbreviations: n= number of subjects, y= years

* Cardiologist, Chiropractor, otorhinolaryngologist, Neurosurgeon, Nurse practitioner, Oncologist, Osteopath, Physical therapist, Rheumatologist, Transplant doctor.

** Neuropsychologist, Oncologist, Psychiatrist/ psychologist

Abbreviations: EMG= electromyography, n.k.= not known

a= Duration of the disease from diagnosis till inclusion in the study

b= Data recorded from the m. tibialis anterior left and/or m. tibialis anterior right

*= Switched the DBS off

Table S3. *Comorbidities*.

Values are given either given in absolute numbers (percentage of total).

Abbreviations: n= number of subjects

Abbreviations: EMG= electromyography, n.k.= not known

a= Duration of the disease from diagnosis till inclusion in the study

b= Data recorded from the m. tibialis anterior left and/or m. tibialis anterior right

*= Switched the DBS off

| Disorder | Total (n=360) | Confirmed EMG (n=147) | Reported EMG (n=213) |
| --- | --- | --- | --- |
| Orthostatic tremor only | 213 (59.2%) | 92 (62.6%) | 121 (56.8%) |
| Anxiety | 80 (22.2%) | 29 (19.7%) | 51 (23.9%) |
| Depression | 58 (16.1%) | 22 (15%) | 36 (16.9%) |
| Essential Tremor | 43 (11.9%) | 12 (8.2%) | 31 (14.6%) |
| Restless Legs Syndrome | 17 (4.7%) | 8 (5.4%) | 9 (4.2%) |
| Ataxia | 7 (1.9%) | 2 (1.4%) | 5 (2.3%) |
| Dystonia | 7 (1.9%) | 4 (2.7%) | 3 (1.4%) |
| Parkinson’s Disease/Parkinsonism | 6 (1.7%) | 4 (2.7%) | 2 (0.9%) |
| Orthostatic Myoclonus | 4 (1.1%) | 3 (2%) | 1 (0.5%) |
| Epilepsy | 4 (1.1%) | 2 (1.4%) | 2 (0.9%) |
| Fibromyalgia | 2 (0.6%) | 1 (0.7%) | 1 (0.5%) |
| Dementia | 1 (0.3%) | 1 (0.7%) |  |
| Multiple Sclerosis | 1 (0.3%) |  | 1 (0.5%) |
| Myasthenia Gravis | 1 (0.3%) | 1 (0.7%) |  |
