# Supplementary material for: Identifying the Diagnostic Challenges and Indicators of Orthostatic Tremor: Patient Perspectives
Source: Mov Disord Clin Pract. 2025 Apr 23;12(8):1124–31. doi: 10.1002/mdc3.70081 (PMC12371454; doi:10.1002/mdc3.70081)
Supplement: Supplementary file 5 — File S1. Includes the web‐based survey titled ‘Symptoms and Diagnosis of Orthostatic Tremor (OT)’ used to collect data. [file MDC3-12-1124-s001.pdf]

1. *INTRODUCTION PAGE*

Thank you for participating in our survey on SYMPTOMS & DIAGNOSIS OF ORTHOSTATIC TREMOR. Your feedback is very important to the OT community. The purpose of these surveys is to help provide information that enables us to understand more about Orthostatic Tremor and share our findings with everyone including the newly-diagnosed as well as the medical community. We are using SurveyMonkey again to conduct this survey. The results are **100% confidential** and the survey can be completed using any type of computer, tablet or smartphone.

The following is a list of sections in the survey, along with the number of questions per section:

1. Introduction Page (0)
2. Qualification Page (1)
3. Demographics & General Background Questions (4)
4. Questions about Symptoms you had before being diagnosed (9)
5. Questions about your original diagnoses (17)
6. Medical Care Questions (7)
7. Questions about Symptoms you had after being diagnosed (4)
8. Follow-up and general questions for comments and feedback (12)

- The survey should take about 30-40 minutes to complete. After you finish each page, you need to hit the 'Next' button to save your progress and continue with the survey. You also have the option to go back to prior pages by hitting the 'Prev' button. If you cannot complete the survey all at once, you can can close it and re-open it from that point by using the link you received via email or a Facebook post.
- The survey will be open approximately 3 weeks. Please try to complete it as soon as possible.
- Although you may have received more than one notification and/or invitation about the survey, please fill out the survey just once.
- If you want to share this survey with another member of the OT community who did not receive the link, you can send us a separate email request at OrthostaticTremorsSurveys@gmail.com, or you can share the Facebook link or forward the email you may have received to them.

Again, thank you for participating in this survey!

2. *QUALIFICATION PAGE*

This survey is intended only for those afflicted with Orthostatic Tremor. For those who have Orthostatic Tremor a assistance with taking the survey, that is acceptable.

*** 1. Have you been diagnosed with Orthostatic Tremor or feel that you have Orthostatic Tremor?**

- ◯ Yes, I have Orthostatic Tremor
- ◯ I feel I have Orthostatic Tremor
- ◯ No, I do not have Orthostatic Tremor but I am taking the survey for someone who has Orthostatic Tremor

3. ***DEMOGRAPHIC & BACKGROUND QUESTIONS***

*** 2. Where is your current primary residence?**

- ◯ Africa
- ◯ Australia
- ◯ Austria
- ◯ Belgium
- ◯ Brazil
- ◯ Canada
- ◯ China
- ◯ England
- ◯ Finland
- ◯ France
- ◯ Germany
- ◯ India
- ◯ Ireland
- ◯ Other (please specify country)
- ◯ Italy
- ◯ Japan
- ◯ Mexico
- ◯ Netherlands
- ◯ New Zealand
- ◯ Norway
- ◯ Philippines
- ◯ Saudi Arabia
- ◯ Scotland
- ◯ Spain
- ◯ Sweden
- ◯ Switzerland
- ◯ United States

*** 3. What is your current age?**

*** 4. Sex**

- ◯ Female
- ◯ Male

*** 5. What is your Race / Ethnicity? (Choose 1 or 2 as it pertains to you)**

- ☐ Hispanic or Latino: a person of Cuban, Mexican, Chicano, Puerto Rican, South or Central American or other Spanish culture or origin, regardless of race
- ☐ White/Caucasian: a person having origins in any of the original peoples of Europe, Middle East, North Africa
- ☐ Asian: a person having origins in any of the original peoples of the Far East, Southeast Asia, or the Indian subcontinent including, for example, Cambodia, China, India, Japan, Korea, Malaysia, Pakistan, the Philippine Islands, Thailand, and Vietnam
- ☐ Black or African American: a person having origins in any of the black racial groups of Africa
- ☐ Native Hawaiian or Other Pacific Islander: a person having origins in any of the original peoples of Hawaii, Guam, Samoa, or other Pacific Islands
- ☐ American Indian or Alaska Native: a person having origins in any of the original peoples of North and South America (including Central America), and who maintains tribal affiliation or community attachment
- ☐ Other: (please specify)

4. ***SYMPTOMS BEFORE DIAGNOSIS***

The following questions will address the symptoms you had before you were diagnosed with Orthostatic Tremor. You will have an opportunity later in the survey to address your current symptoms/severity.

* 6. At what age do you feel your Orthostatic Tremors started? (Please estimate if not sure)

* 7. What symptoms did you have that made you go see a doctor before being diagnosed with Orthostatic Tremor? (Each symptom requires a response)

	Yes	No	Sometimes
I had to lean against something while cooking or doing other chores for stability	○	○	○
I had the feeling of unsteadiness or imbalance	○	○	○
I felt like I was going to fall when standing	○	○	○
I felt like I was going to fall when walking	○	○	○
My leg(s) locked while standing	○	○	○
My legs(s) felt heavy	○	○	○
I had to stand on one leg/foot at a time, switching often (like a stork)	○	○	○
I found it difficult to move along in a line side to side, such as a receiving line or a buffet	○	○	○
I had to "curl" my toes for extra support in order to stand	○	○	○
I had to "rock" back and forth on each leg in order to stand	○	○	○
I experienced shortness of breath while standing	○	○	○
I was unable to walk without assistance	○	○	○
I fell when standing	○	○	○
I fell when walking	○	○	○
The shakiness/tremors disappeared partially or completely when I walked or sat	○	○	○

*** 7. CONTINUED - What symptoms did you have that made you go see a doctor before being diagnosed with Orthostatic Tremor? (Each symptom requires a response)**

	Yes	No	Sometimes
I felt that the shakiness/tremors were more intense when standing in a small enclosed space (e.g. a shower stall)	◯	◯	◯
The shakiness/tremors affected both legs	◯	◯	◯
The shakiness/tremors affected both arms	◯	◯	◯
Others pointed out that they could see/feel the shakiness/tremors in my leg(s)	◯	◯	◯
Others pointed out that they could see/feel the shakiness/tremors in my arm(s)	◯	◯	◯
The shakiness/tremors seemed worse when I was tired or physically exhausted	◯	◯	◯
The shakiness/tremors seemed worse when I was not feeling well	◯	◯	◯
I felt my heart racing/palpitating when standing	◯	◯	◯
I had difficulty writing or had shaky handwriting	◯	◯	◯
I experienced a 'rushing sensation' in my legs and/or arms, similar to an adrenaline rush	◯	◯	◯
I noticed (or had been told) that I slurred my speech	◯	◯	◯
Stress (or strong emotions) seemed to trigger my symptoms	◯	◯	◯
I felt anxious	◯	◯	◯
I felt nauseous	◯	◯	◯

4. SYMPTOMS BEFORE DIAGNOSIS - Continued

* 8. Please rate the severity of the symptoms you had that made you go see a doctor before being diagnosed with Orthostatic Tremor. (Each symptom requires a response)

	Not a problem	Moderate problem	Challenging	Severe
I had shakiness/tremors in my leg(s) that occurred when standing	○	○	○	○
I had trouble standing in a line/queue	○	○	○	○
I had trouble shopping in a store	○	○	○	○
I experienced pain/cramps in my legs	○	○	○	○
I had muscular leg stiffness	○	○	○	○
My leg(s) felt weak	○	○	○	○
I had shakiness/tremors in my arm(s) when my arm(s) were extended away from my torso (e.g., reaching for something)	○	○	○	○
I had shakiness/tremors in my arm(s) when I leaned on them (weight-bearing)	○	○	○	○
I experienced shakiness/tremors in my arm when holding a fork or cup	○	○	○	○
My arm(s) felt weak	○	○	○	○

9. As a follow-up to the previous two questions, if you had any additional symptoms as related to Orthostatic Tremor that are not included above on why you went to see a doctor before you were diagnosed with Orthostatic Tremor, please describe and indicate the severity (M, C or S) in the box below for each individual symptom (if applicable).

Additional Symptom #1

Symptom #1: M=Moderate, C=Challenging, S=Severe

Additional Symptom #2

Symptom #2: M=Moderate, C=Challenging, S=Severe

Additional Symptom #3

Symptom #3: M=Moderate, C=Challenging, S=Severe

Additional Symptom #4

Symptom #4: M=Moderate, C=Challenging, S=Severe

*** 10. The following two questions pertain to standing before being diagnosed:**

	N/A	< 30 seconds	between 30 seconds and 1 minute	between 2 - 5 minutes	more than 5 minutes
On average, how long did it take for the shakiness/tremors to begin upon standing?	◯	◯	◯	◯	◯
If you "curled" your toes for extra support when standing, how soon after standing?	◯	◯	◯	◯	◯

*** 11. Have you experienced a non-diagnosed trauma to the head or neck prior to the appearance of Orthostatic Tremor symptoms? If yes, please indicate how many.**

◯ No, I have not had a non-diagnosed trauma

◯ 1

◯ 2

◯ 3

◯ More than 3

*** 12. Have you ever had a diagnosed concussion prior to the appearance of Orthostatic Tremor symptoms? If yes, please indicate how many.**

◯ No, I have not had a diagnosed concussion

◯ 1

◯ 2

◯ 3

◯ More than 3

*** 13. Have you ever experienced any of the following prior to the appearance of Orthostatic Tremor symptoms? (Choose all that apply)**

☐ Whiplash (trauma/injury caused by a severe jerk to the head)

☐ Chiropractic treatment on the neck

☐ Brain surgery

☐ Neck surgery

☐ Upper back/spinal surgery

☐ Stroke

☐ Brain radiation therapy

☐ Chemo

☐ None of the above

☐ Other trauma to the head, neck or spine (please specify)

4. *SYMPTOMS BEFORE DIAGNOSIS - Continued*

* 14. Did you experience an illness/traumatic incident that you feel might have triggered the onset of Orthostatic Tremor?

- ◯ No
- ◯ Yes (please specify)

5. *DIAGNOSIS QUESTIONS*

* 15. What age were you when you first went to see a doctor about your symptoms as related to your Orthostatic Tremor? (Please estimate if not sure). If you are still waiting to see a doctor, just enter 0.

* 16. Which medical professional did you first visit to discuss your symptoms as related to your Orthostatic Tremor?

- ◯ I have not seen a doctor yet
- ◯ General Practitioner (Primary Care Physician)
- ◯ Neurologist (General)
- ◯ Neurologist (Movement Disorder Specialist)
- ◯ Osteopath
- ◯ Physiatrist
- ◯ Physiologist
- ◯ Psychologist/Psychiatrist
- ◯ Other (please specify)

* 17. When you first went to this doctor for your symptoms, what was your original diagnosis? Choose all those that may apply if you were told that you had more than one condition on your first visit.

- ☐ I have not seen a doctor yet
- ☐ I was told there was nothing neurologically wrong
- ☐ The doctor did not know what was wrong
- ☐ Orthostatic Tremor
- ☐ Alzheimer's Disease (AD) and other dementias
- ☐ ALS (Amyotrophic Lateral Sclerosis)
- ☐ Anxiety
- ☐ Mental/Psychological
- ☐ Ataxia
- ☐ Dystonia
- ☐ Epilepsy
- ☐ Essential Tremor (ET)
- ☐ Huntington's Disease
- ☐ Hypoglycemia
- ☐ Multiple Sclerosis (MS)
- ☐ Myasthenia Gravis
- ☐ Orthostatic Myoclonus (OM)
- ☐ Parkinson's/Parkinson's-related
- ☐ Restless Leg Syndrome
- ☐ Other (please specify your first diagnosis/diagnoses if NOT listed above)

*** 18. Please indicate all other diagnoses that you received from any other medical professionals prior to getting the actual diagnosis of Orthostatic Tremor (Choose all that apply)**

- [ ] I have not seen a doctor yet
- [ ] I was diagnosed with Orthostatic Tremor on my first visit
- [ ] I have not been officially diagnosed with Orthostatic Tremor
- [ ] I was told there was nothing neurologically wrong
- [ ] The doctor did not know what was wrong
- [ ] Alzheimer's Disease (AD) and other dementias
- [ ] ALS (Amyotrophic Lateral Sclerosis)
- [ ] Anxiety
- [ ] Mental/Psychological
- [ ] Ataxia
- [ ] Dystonia
- [ ] Epilepsy
- [ ] Essential Tremor (ET)
- [ ] Huntington's Disease
- [ ] Hypoglycemia
- [ ] Multiple Sclerosis (MS)
- [ ] Myasthenia Gravis
- [ ] Orthostatic Myoclonus (OM)
- [ ] Parkinson's/Parkinson's-related
- [ ] Restless Leg Syndrome
- [ ] Other (please specify any other diagnoses if NOT listed above)

*** 19. Did you feel that you had Orthostatic Tremor before a medical professional diagnosed you?**

- ○ No
- ○ Yes, but I did not tell them
- ○ Yes, and I told them and they agreed
- ○ Yes, and I told them and they disagreed
- ○ Yes, I believe I have Orthostatic Tremor but I have yet to be officially diagnosed

*** 20. How many different medical professionals did you have to see before getting an official diagnosis of Orthostatic Tremor? Include in your count the medical professional who diagnosed you (Please estimate if not sure)**

- [ ] I have not been officially diagnosed
- [ ] 1
- [ ] 2
- [ ] 3
- [ ] 4
- [ ] 5
- [ ] 6
- [ ] 7
- [ ] 8 or more

*** 21. Which of the following medical professionals officially diagnosed you with Orthostatic Tremor?**

- ○ I have not been officially diagnosed
- ○ General Practitioner (Primary Care Physician)
- ○ Neurologist (General)
- ○ Neurologist (Movement Disorder Specialist)
- ○ Osteopath
- ○ Physiatrist
- ○ Physiologist
- ○ Psychologist/Psychiatrist
- ○ Other medical professional (please specify)

5. *DIAGNOSIS QUESTIONS - Continued*

* 22. Where were you originally diagnosed with Orthostatic Tremor?

- ◯ Not Diagnosed
- ◯ Africa
- ◯ Australia
- ◯ Austria
- ◯ Belgium
- ◯ Brazil
- ◯ Canada
- ◯ China
- ◯ England
- ◯ Finland
- ◯ France
- ◯ Germany
- ◯ India
- ◯ Ireland
- ◯ Other (please specify country)
- ◯ Italy
- ◯ Japan
- ◯ Mexico
- ◯ Netherlands
- ◯ New Zealand
- ◯ Norway
- ◯ Philippines
- ◯ Saudi Arabia
- ◯ Scotland
- ◯ Spain
- ◯ Sweden
- ◯ Switzerland
- ◯ United States

* 23. If you have been officially diagnosed with Orthostatic Tremor, what age were you when you were diagnosed? (Please estimate if not sure). If you have not been officially diagnosed, just enter 0.

* 24. As of today, what neurological disorders have you been officially diagnosed with? (Choose all that apply)

- ☐ None
- ☐ Orthostatic Tremor
- ☐ Anxiety
- ☐ Depression
- ☐ Mental/Psychological
- ☐ Alzheimer's Disease (AD) and other dementias
- ☐ ALS (Amyotrophic Lateral Sclerosis)
- ☐ Ataxia
- ☐ Cerebellar Tremor
- ☐ Other (please specify neurological disorder if NOT listed above)
- ☐ Dystonia
- ☐ Epilepsy
- ☐ Essential Tremor (ET)
- ☐ Huntington's Disease (HD)
- ☐ Multiple Sclerosis (MS)
- ☐ Myasthenia Gravis (MG)
- ☐ Orthostatic Myoclonus (OM)
- ☐ Parkinson's Disease (PD) or PD-related disorders
- ☐ Restless Leg

5. ***DIAGNOSIS QUESTIONS - Continued***

*** 25. Do/did you have any other immediate family member who has/had a neurological-diagnosed disorder? This would be a "blood relative" (e.g., parents, grandparents, siblings, children, grandchildren). If so, choose all neurological disorders that apply.**

- ☐ None that I'm aware of
- ☐ Orthostatic Tremor
- ☐ Alzheimer's Disease (AD) and other dementias
- ☐ ALS (Amyotrophic Lateral Sclerosis)
- ☐ Dystonia
- ☐ Epilepsy
- ☐ Essential Tremor (ET)
- ☐ Huntington's Disease (HD)
- ☐ Multiple Sclerosis (MS)
- ☐ Myasthenia Gravis (MG)
- ☐ Orthostatic Myoclonus (OM)
- ☐ Parkinson's Disease (PD) or PD-related disorders
- ☐ Restless Leg
- ☐ Other (please specify the neurological disorder if NOT listed above)

26. Per the previous question, if you have other family members with a neurological disorder, can you be more specific? If any family member also has OT, please share any similarities/differences in symptoms, etc.

Father	
Mother	
Sibling	
Children	
Grandchildren	
Grandparent	

*** 27. Are you a multiple birth? (Choose all that apply)**

- ☐ No
- ☐ Yes, Identical
- ☐ Yes, Fraternal
- ☐ Yes, they had/have OT also
- ☐ Yes, they do/did not have OT
- ☐ Yes, they had/have other neurological issues
- ☐ If you answered Yes to any of the above, please share any additional information as it pertains to OT (e.g., are you a twin/triplet, are they M/F, are they alive or deceased, etc)

5. *DIAGNOSIS QUESTIONS - Continued*

*** 28. Have you ever had a Electromyography (EMG) as related to your Orthostatic Tremor? (Choose all that apply)**

- An EMG measures muscle response or electrical activity in response to a nerve's stimulation of the muscle. The test is used to help detect neuromuscular abnormalities. During the test, one or more small needles (also called electrodes) are inserted through the skin into the muscle.

- [ ] No
- [ ] I don't know
- [ ] Yes, while standing
- [ ] Yes, while sitting
- [ ] Yes, while lying down
- [ ] If Yes, please share the results (e.g., Results came back normal while lying down, not normal while standing, etc)

*** 29. Have you ever had a Nerve Conduction Velocity (NCV) test — also called a Nerve Conduction Study (NCS) as related to your Orthostatic Tremor? (Choose all that apply)**

- This test measures how fast an electrical impulse moves through your nerve. NCV can identify nerve damage. During this type of test, your nerve is stimulated, usually with electrode patches attached to your skin.

- [ ] No
- [ ] I don't know
- [ ] Yes, while standing
- [ ] Yes, while sitting
- [ ] Yes, while lying down
- [ ] If Yes, please share the results (e.g., Results came back normal for legs, not normal for arms, etc)

*** 30. Have you had a Magnetic Resonance Imaging (MRI) scan of your brain as related to your Orthostatic Tremor? (Choose all that apply)**

- This test is a medical imaging technique used in radiology to form pictures of the anatomy and the physiological processes of the body in both health and disease. MRI scanners use strong magnetic fields, magnetic field gradients, and radio waves to generate images of the organs in the body.

- [ ] No
- [ ] I don't know
- [ ] Yes
- [ ] If Yes, please share any feedback you received on your MRI

*** 31. Have you had a "Frequency Test" of the Orthostatic Tremors on your legs? (Choose all that apply)**

- This is a simple test where you or your doctor can measure the tremor frequency on your legs while sitting and while standing. A device such as a smart phone with an application that measures the frequency of the tremors can also be used (as seen in the picture). The 'sitting' result should be compared to the 'standing' result, with the 'standing' results coming in at a much higher Hz (13-20Hz).

- ☐ No
- ☐ Yes, by General Practitioner (Primary Care Physician)
- ☐ Yes, by Neurologist (General)
- ☐ Yes, by Neurologist (Movement Disorder Specialist)
- ☐ Yes, by an Osteopath
- ☐ Yes, by a Physiatrist
- ☐ Yes, by a Physiologist
- ☐ Yes, on my own
- ☐ I don't know
- ☐ If Yes, or by another medical professional, please share results (e.g., If you remember any results such as standing was higher than lying down, with a measurement somewhere around 13-20 hz, etc)

Example of "Frequency Test" using the iPhone app "Lift Pulse" (Note: This is no longer available if you did not install it on your iPhone last year. It is available for Android devices for free)

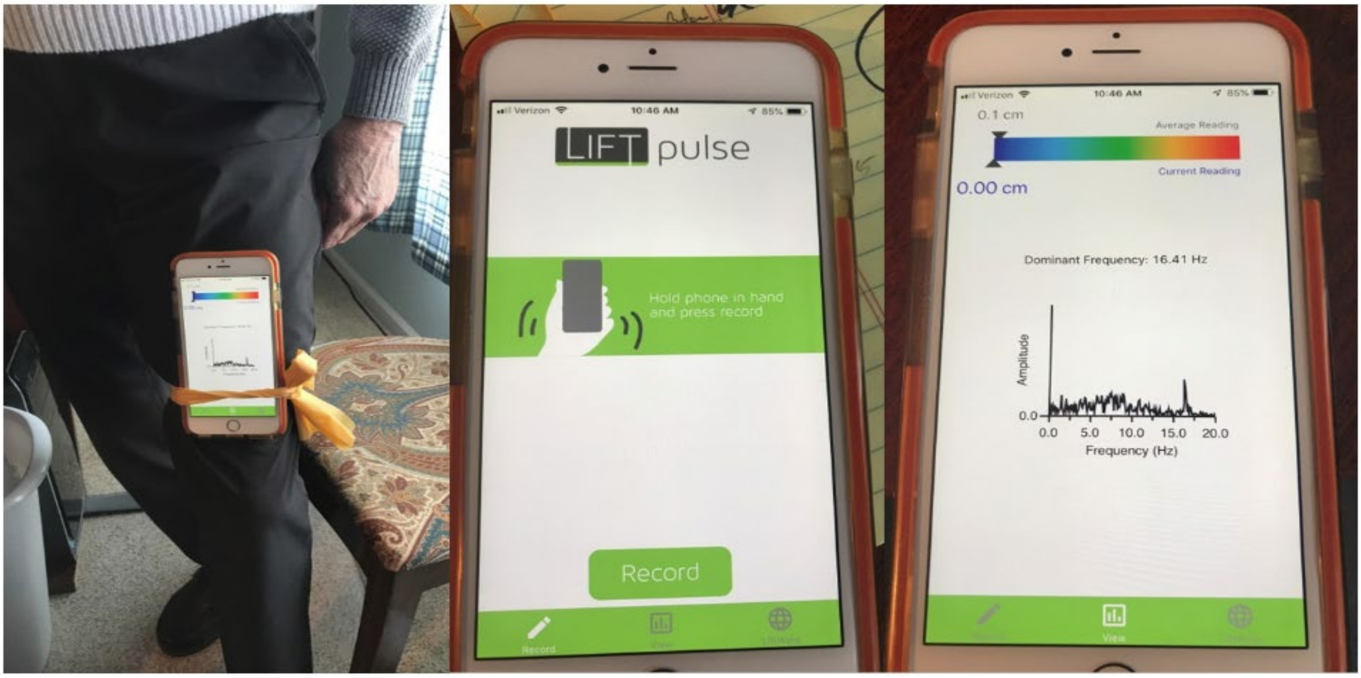

5. DIAGNOSIS QUESTIONS - Continued

The following is an app that is available for the Apple iPhone. It is $3.99. It takes measurements for a full minute, but for those who can not stand that long, if you gently sit down after your tremors have started, the reading should be the same. This app does a nice job in tracking your tremors over time. After saving your "test", you can go back and click on the calendar and review past results.

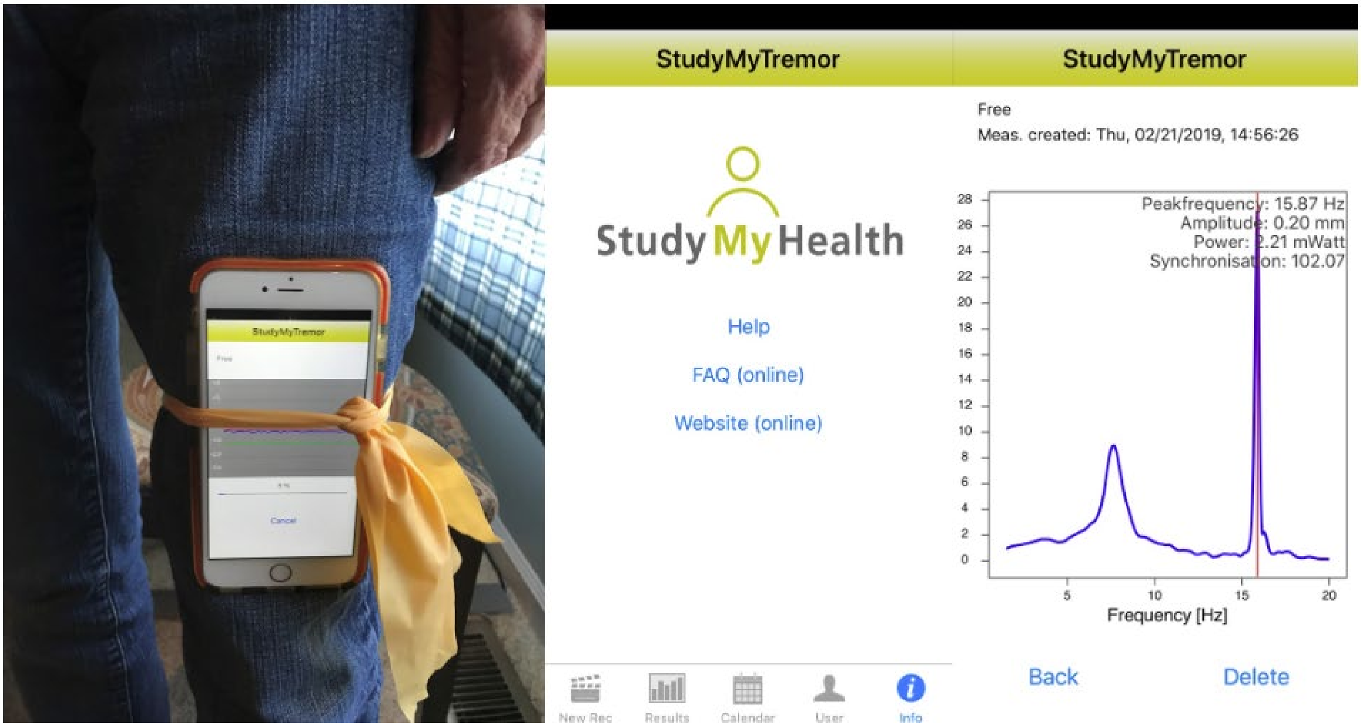

6. MEDICAL CARE QUESTIONS

*** 32. The following four questions pertain to experiencing any tremors in your eyes:**

	Yes	No	N/A
Have you mentioned to your eye doctor that you have Orthostatic Tremor?	○	○	○
Did your doctor then check to see if your eyes tremor when standing?	○	○	○
Did your eyes tremor?	○	○	○
Did the doctor mention if he/she has seen that before?	○	○	○

*** 33. Who prescribes your medications for Orthostatic Tremor? (Choose all that apply)**

- ☐ I don't take medications for Orthostatic Tremor
- ☐ General Practitioner
- ☐ Neurologist (General)
- ☐ Neurologist (Movement Disorder Specialist)
- ☐ Other (please specify)
- ☐ Osteopath
- ☐ Physiatrist
- ☐ Psychologist/Psychiatrist

*** 34. Over the years how many neurologists would you say you have seen as related to your Orthostatic Tremor? (Please estimate if not sure)**

- ◯ None
- ◯ 1
- ◯ 2
- ◯ 3
- ◯ 4
- ◯ 5
- ◯ 6
- ◯ 7
- ◯ 8 or more

*** 35. Do you currently have a neurologist that you see for Orthostatic Tremor? (Choose all that apply)**

- ☐ Yes, I have one
- ☐ No, I do not have one
- ☐ Searching for a new one
- ☐ I see my General Practitioner (Primary Care Physician) for Orthostatic Tremor
- ☐ Other (please specify)

*** 36. How happy are you with your current (or most-recently visited) neurologist?**

- ◯ Very happy
- ◯ Somewhat happy
- ◯ Not at all happy
- ◯ I currently don't have a neurologist

*** 37. If you have a neurologist, when was the last time you saw her/him as it pertains to Orthostatic Tremor?**

- ◯ I don't have a neurologist
- ◯ Less than 6 months ago
- ◯ Between 6 months and 1 year
- ◯ Between 1 year and 2 years
- ◯ More than 2 years ago

*** 38. Was your current / most recent neurologist aware of Orthostatic Tremor when you first went to see her/him, or did you have to explain it?**

- ◯ The doctor was not aware
- ◯ The doctor was aware of Orthostatic Tremor, but was not very knowledgeable about it
- ◯ The doctor had extensive knowledge of Orthostatic Tremor
- ◯ Not sure
- ◯ I have yet to be diagnosed with Orthostatic Tremor
- ◯ I don't have a neurologist
- ◯ Other (please specify)

7. SYMPTOMS AFTER DIAGNOSIS

The following questions will address the symptoms you have since being diagnosed with Orthostatic Tremor

*** 39. What symptoms do you currently have as related to Orthostatic Tremor? (Each symptom requires a response)**

	Yes	No	Sometimes
I have to lean against something while cooking or doing other chores for stability	○	○	○
I have the feeling of unsteadiness or imbalance	○	○	○
I feel like I am going to fall when standing	○	○	○
I feel like I am going to fall when walking	○	○	○
My leg(s) lock while standing	○	○	○
My legs(s) feel heavy	○	○	○
I stand on one leg/foot at a time switching often (like a stork)	○	○	○
I find it difficult to move along in a line side to side, such as a receiving line or a buffet	○	○	○
I have to "curl" my toes for extra support in order to stand	○	○	○
I have to "rock" back and forth on each leg in order to stand	○	○	○
I experience shortness of breath while standing	○	○	○
I am unable to walk without assistance	○	○	○
I fall when standing	○	○	○
I fall when walking	○	○	○
The tremors disappear partially or completely when I walk or sit	○	○	○
I feel that the tremors are more intense when standing in a small enclosed space (e.g. a shower stall)	○	○	○
The tremors affect both legs	○	○	○
The tremors affect both arms	○	○	○
Others have pointed out that they can see/feel the tremors in my leg(s)	○	○	○

*** 39. CONTINUED - What symptoms do you currently have as related to Orthostatic Tremor? (Each symptom requires a response)**

	Yes	No	Sometimes
Others have pointed out that they can see/feel the tremors in my leg(s)	○	○	○
Others have pointed out that they can see/feel the tremors in my arm(s)	○	○	○
The tremors seem worse when I am tired or physically exhausted	○	○	○
The tremors seem worse when I am not feeling well	○	○	○
I feel my heart racing/palpitating when standing	○	○	○
I have difficulty writing or have shaky handwriting	○	○	○
I experience a 'rushing sensation' in my legs and/or arms, similar to an adrenaline rush	○	○	○
I notice (or have been told) that I slur my speech	○	○	○
Stress (or strong emotions) seem to trigger my symptoms	○	○	○
I feel anxious	○	○	○
I feel nauseous	○	○	○

* 40. Please rate the severity of the symptoms you currently have as related to Orthostatic Tremor. (Each symptom requires a response)

	Not a problem	Moderate problem	Challenging	Severe
I have rapid tremors/vibrations in my leg(s) that occur when standing	○	○	○	○
I have trouble standing in a line/queue	○	○	○	○
I have trouble shopping in a store	○	○	○	○
I experience pain/cramps in my legs	○	○	○	○
I have muscular leg stiffness	○	○	○	○
My leg(s) feel weak	○	○	○	○
I have tremors in my arm(s) when my arm(s) are extended away from my torso (e.g., reaching for something)	○	○	○	○
I have tremors in my arm(s) when I lean on them (weight-bearing)	○	○	○	○
I experience the tremor in my arm when holding a fork or cup	○	○	○	○
My arm(s) feel weak	○	○	○	○

41. As a follow-up to the previous two questions, if you have any additional symptoms as related to Orthostatic Tremor that are not included above, please describe and indicate the severity (M, C or S) in the box below for each individual symptom (if applicable).

Additional Symptom #1 ______

Symptom #1: M=Moderate, C=Challenging, S=Severe ______

Additional Symptom #2 ______

Symptom #2: M=Moderate, C=Challenging, S=Severe ______

Additional Symptom #3 ______

Symptom #3: M=Moderate, C=Challenging, S=Severe ______

Additional Symptom #4 ______

Symptom #4: M=Moderate, C=Challenging, S=Severe ______

7. *SYMPTOMS AFTER DIAGNOSIS - Continued*

* **42. The following two questions pertain to your current ability when standing:**

	N/A	< 30 seconds	between 30 seconds and 1 minute	between 2 - 5 minutes	more than 5 minutes
On average, how long does it take for the leg tremors to begin upon standing?	○	○	○	○	○
If you "curl" your toes for extra support when standing, how soon after standing?	○	○	○	○	○

8. *FOLLOW-UP & GENERAL QUESTIONS*

* **43. Did you participate in the first survey we conducted in April of 2018 about Prescribed Medications and Alternative Therapies?**

- ○ Yes
- ○ No - I was not diagnosed with OT at that time
- ○ No - I was not aware of it
- ○ No - I opted not to participate
- ○ Other (please specify)

* **44. Have you seen and reviewed the survey summary and presentation? It can be found on the following website: https://www.orthostatictremor.org/files/OT_Survey_April2018v01.pdf (you can copy and paste this address)**

- ○ Yes
- ○ No
- ○ No, but I plan to review it

* **45. Have you shared the presentation or any parts of it with any of your doctors? (Choose all that apply)**

- ☐ No, I did not
- ☐ No, I was not aware of the study
- ☐ No, but I plan to tell them about it
- ☐ Yes, with General Practitioner (Primary Care Physician)
- ☐ Yes, with Neurologist (General)
- ☐ Yes, with Neurologist (Movement Disorder Specialist)
- ☐ Yes, with Osteopath
- ☐ Yes, with Physiatrist
- ☐ Yes, with Psychologist/Psychiatrist
- ☐ Yes, with another medical professional (please specify in feedback textbox below)
- ☐ Can you share any feedback that your doctor(s) had?

8. *FOLLOW-UP & GENERAL QUESTIONS - Continued*

*** 46. If you did review the first survey, did you try anything new that you saw in it (e.g., exercise, medications, essential oils...)? (Choose all that apply)**

- ☐ I did not review the first survey
- ☐ No
- ☐ Yes
- ☐ If yes, can you share what you tried, as well as the outcome?

*** 47. Are you willing to participate in the next survey that we plan to do? It will focus on Coping Skills and Assistive Devices (Choose all that apply)**

- ☐ Yes
- ☐ I don't know yet
- ☐ No
- ☐ If not, can you let us know why?

48. Do you know of any current or upcoming research studies on Orthostatic Tremor? (Choose all that apply)

- ☐ I am not aware of any research studies
- ☐ Yes
- ☐ Yes, I plan to participate
- ☐ If yes, can you share any information about the studies and where they are being conducted?

49. How do you spread the word about Orthostatic Tremor? (Choose all that apply)

- ☐ Family
- ☐ Extended family
- ☐ Friends
- ☐ Social media
- ☐ General Practitioner (Primary Care Physician)
- ☐ Neurologist
- ☐ Other medical professionals
- ☐ Other (please specify)

9. *Last 5 Questions*

50. Do you visit or participate in any Orthostatic Tremor sites? (Choose all that apply)

- ☐ No
- ☐ Yes, website
- ☐ Yes, Facebook
- ☐ Other (please specify)

9. ***Last 5 Questions - Continued***

The following is an image of some known OT Facebook groups and website pages that we will publish in the final presentation along with any additional Facebook and web pages you might know of and wish to share.

Please scroll past this image and answer the final four questions, then hit DONE to complete the survey!

Web Address or FaceBook Look Up	Facebook Web Address	Members or Likes	Oldest Post	Home Country
**Orthostatic Tremor** (Stared in US and Canada Open to All)	https://www.facebook.com/orthostatictremor/	785	Mar-2000	United States and Canada
**Primary Orthostatic Tremor** (Started in Australia - Open to All)	https://www.facebook.com/groups/orthostatic.tremor/?ref=group_header	533	May-2011	Australia
**Fycompa and OT**	https://www.facebook.com/groups/464612497281739/?ref=group_header	196	Aug-2018	Multi-National
**Orthostatische Tremor / Primary Orthostatic Tremor** (Started in Netherlands - Closed Group)	https://www.facebook.com/groups/246769345421862/?ref=group_header	173	Apr-2012	Nietherlands
**RareConnect** (Started in France - Open to all - Has Dedicated OT Webpage see below)	https://www.facebook.com/rareconnect/	121	Jan-2009	Paris, France
**Trillende benen bij stilstaan - OT Nederland - Orthostatische Tremor**	https://www.facebook.com/OTNederland/	113	Aug-2016	Nederland (Netherlands)
**Orthostatic Tremor Awareness**	https://www.facebook.com/Shakylegsyndrom/	93	Jan-2019	United States
**Orthostatic Tremor: Am I The Only One**	https://www.facebook.com/228872750624595/	73	Feb-2016	
**Orthostatic Tremors**	https://www.facebook.com/groups/401694923308983/?ref=group_header	72	Jan-2014	United States
**Orthostatic Tremor Friends**	https://www.facebook.com/698183083590834/photos/698185030257306/	64	Oct-2014	Multi-National
**TREMBLEMENT ORTHOSTATIQUE PRIMAIRE (SUITE)** (This is a "secret group", must message Admin to ask to join)	https://www.facebook.com/groups/CloEdith/?ref=group_header	56	Jun-2016	France
**National Tremor Foundation**	https://www.facebook.com/NationalTremorFoundation/	1638	1994	United Kingdom
**National Tremor Foundation Group**	https://www.facebook.com/groups/624447414609460/?ref=group_header	1591	1994	United Kingdom

Name	AKA	Facebook Address if known	About Page Comments
Orthostatic Tremors	https://www.orthostatictremor.org/	https://www.facebook.com/orthostatictremor/	Dedicated to OT
RareConnect	https://www.rareconnect.org/en/community/primary-orthostatic-tremor	https://www.facebook.com/rareconnect/	
National Institite of Health - (NIH)	https://www.nih.gov/		The (NIH), a part of the U.S. Department of Health and Human Services, is the nation's medical research agency.
Clinical Trials Database - (NIH)	https://clinicaltrials.gov/ct2/home		Dedicated to NIH Clinical Trails
National Tremor Foundation	https://tremor.org.uk/		Seems mostly dedicatet to ET?
NORD (National Organization for Rare Disorders)	https://rarediseases.org/		NORD, is a patient advocacy organization dedicated to individuals with rare diseases. With its more than 280 patient organization members, is committed to the identification, treatment, and cure of rare disorders through programs of education, advocacy, research, and patient services.
Orphanet Journal of Rare Diseases	https://ojrd.biomedcentral.com/		Orphanet Journal of Rare Diseases is an open access, peer-reviewed journal that encompasses all aspects of rare diseases and orphan drugs. The journal publishes high-quality reviews on specific rare diseases. In addition, the journal may consider articles on clinical trial outcome reports, either positive or negative, and articles on public health issues in the field of rare diseases and orphan drugs.
Association of People Affected by Essential Tremor - (APTES)	https://www.aptes.org/tremblement-essentiel/tremblement-orthostatique/		Essential tremor is a neurological and genetic disease. This is the most common cause of tremors. It affects 1 in 200 people . It concerns both women and men and worsens over time. Aptes.org provides information on essential tremor and the course of care.

51. Please share any other OT websites, Twitter Feeds, Blogs, Facebook groups or other social media sites that we may have missed.

Additional Sites

Additional Sites

Additional Sites

Additional Sites

Additional Sites

Additional Sites

Additional Sites

52. Can you share with us how you first heard about this survey?

- ○ https://www.orthostatictremor.org webpage
- ○ A forwarded communication from a friend
- ○ Facebook Page (please specify below)
- ○ Other (please share)

53. We know that there were a lot of questions in this survey. Please use the slider to rate how you felt about the number of questions.

# of questions was okay	# was a lot, but I see the need	Too many questions

54. Please feel free to share any comments/thoughts you have with the Orthostatic Tremor community.
(Thank you again for your participation. A presentation of the survey results will be created and shared via the various websites and FB pages.)

- ☐ I do not have any feedback at this time
- ☐ Please share any comments/thoughts
