# Supplementary material for: Identifying the Diagnostic Challenges and Indicators of Orthostatic Tremor: Patient Perspectives
Source: Mov Disord Clin Pract. 2025 Apr 23;12(8):1124–31. doi: 10.1002/mdc3.70081 (PMC12371454; doi:10.1002/mdc3.70081)
Supplement: Supplementary file 6 — Table S1. Patient Demographics. [file MDC3-12-1124-s005.docx]

Table S1. *Patient Demographics*.

Values are given either given in absolute numbers (percentage of total), unless otherwise indicated.

Abbreviations: n= number of subjects, y= years

*Africa, Belgium, Brazil, Channel Islands, Ireland, Isle of Man, Israel, Italy, Luxembourg, Mexico, New Zealand, Northern Ireland, Norway, Scotland, South Africa, Sweden, Switzerland, Wales.

Abbreviations: EMG= electromyography, n.k.= not known

a= Duration of the disease from diagnosis till inclusion in the study

b= Data recorded from the m. tibialis anterior left and/or m. tibialis anterior right

*= Switched the DBS off

| Characteristics | | Total (n=360) | Confirmed EMG (n=147) | Reported EMG (n=213) |
| --- | --- | --- | --- | --- |
| Gender (n) | Male | 73 (20.3%) | 27 (18.4%) | 46 (21.6) |
|  | Female | 287 (79.7%) | 120 (81.6%) | 167 (78.4) |
| Age (y, range) | | 66.4 (range 33-90) | 65.5 (39-90) | 67.1 (33-86) |
| Ethnicity (n) | Caucasian | 350 (97.1%) | 142 (96.6%) | 207 |
|  | Hispanic | 6 (1.7%) | 3 (2%) | 3 |
|  | Black | 2 (0.6%) | 1 (0.7%) | 1 |
|  | Asian | 2 (0.6%) | 1 (0.7%) | 1 |
| Country of Residence (n) | United States | 161 (44.7%) | 48 (32.7%) | 113 |
|  | United Kingdom | 43 (11.9%) | 22 (15%) | 22 |
|  | The Netherlands | 38 (10.6%) | 21 (14.3%) | 21 |
|  | Australia | 34 (9.4%) | 12 (8.2%) | 17 |
|  | Canada | 29 (8.1%) | 12 (8.2%) | 17 |
|  | France | 25 (6.9%) | 20 (13.6%) | 5 |
|  | Others* | 30 (8.3%) | 12 (8.2%) | 18 |
