# Supplementary material for: Identifying the Diagnostic Challenges and Indicators of Orthostatic Tremor: Patient Perspectives
Source: Mov Disord Clin Pract. 2025 Apr 23;12(8):1124–31. doi: 10.1002/mdc3.70081 (PMC12371454; doi:10.1002/mdc3.70081)
Supplement: Supplementary file 7 — Table S2. Diagnostic Characteristics. [file MDC3-12-1124-s002.docx]

Table S2. *Diagnostic Characteristics*.

Abbreviations: EMG= electromyography, n.k.= not known

a= Duration of the disease from diagnosis till inclusion in the study

b= Data recorded from the m. tibialis anterior left and/or m. tibialis anterior right

*= Switched the DBS off

| Characteristics | | Total (n=360) | Confirmed EMG (n=147) | Reported EMG (n=213) |
| --- | --- | --- | --- | --- |
| Age of onset (y) | | 51.3 (8-80) | 50.3 (8-70) | 52.1 (16-80) |
| Age at diagnosis (y) | | 59 | 57.7 (30-74) | 59.9 (28-82) |
| Diagnostic delay (y) | Total |  |  |  |
|  | # of years before discussing with physician | 3.9 (1-40) | 3.4 (1-27) | 4.1 (0-40) |
|  | # of years to receive OT diagnosis after first speaking with physician | 4.1 ( 0-50) | 4.3 (1-50) | 4.0 (0-38) |
| # of physicians seen before receiving diagnosis (n) | | 3 (1-8+) | 3.2 (1-8+) | 2.9 (1-8+) |
| Practitioner symptoms first discussed with (n) | General Practitioner | 237 (65.8%) | 93 (63.3%) | 144 (67.6%) |
|  | General Neurologist | 74 (20.6%) | 32 (21.8%) | 42 (19.7%) |
|  | Movement Disorders Neurologist | 26 (7.2%) | 7 (4.8%) | 19 (8.9%) |
|  | Orthopedics | 6 (1.7%) | 3 (2.0%) | 3 (1.4%) |
|  | Psychiatrist/ psychologist | 5 (1.4%) | 4 (2.7%) | 1 (0.5%) |
|  | Other* | 12 (3.3%) | 8 (5.4%) | 4 (1.9%) |
| Diagnoses received prior to OT | Orthostatic tremor on first visit | 52 (14.4%) | 20 (13.6%) | 32 (15%) |
|  | No diagnosis on 1^st^ visit | 108 (30%) | 64 (43.5%) | 44 (20.7%) |
|  | “Nothing is wrong” | 14 (3.9%) | 6 (4.1%) | 8 (3.8%) |
|  | Essential tremor | 36 (10%) | 12 (8.2%) | 24 (11.2%) |
|  | Mental/psychological | 39 (10.8%) | 25 (17%) | 14 (6.6%) |
|  | Anxiety | 42 (11.6%) | 22 (15%) | 20 (9.4%) |
|  | PD | 17 (4.7%) | 8 (5.4%) | 9 (4.2%) |
|  | Spinal disorder | 8 (2.2%) | 2 (1.4%) | 6 (2.8%) |
|  | RLS | 10 (2.7%) | 7 (4.7%) | 3 (1.4%) |
| Practitioner that diagnosed OT (n) | General Practitioner | 5 (1.4%) | 2 (1.4%) | 3 (1.4%) |
|  | General Neurologist | 185 (51.4) | 69 (46.9%) | 116 (54.5%) |
|  | Movement Disorders Neurologist | 167 (46.4%) | 75 (51%) | 92 (43.2%) |
|  | Other** | 3 (0.8%) | 1 (0.7%) | 2 (0.9%) |

Values are given either given in average number (range) or in absolute numbers (percentage of total).

Abbreviations: n= number of subjects, y= years

* Cardiologist, Chiropractor, otorhinolaryngologist, Neurosurgeon, Nurse practitioner, Oncologist, Osteopath, Physical therapist, Rheumatologist, Transplant doctor.

** Neuropsychologist, Oncologist, Psychiatrist/ psychologist

Abbreviations: EMG= electromyography, n.k.= not known

a= Duration of the disease from diagnosis till inclusion in the study

b= Data recorded from the m. tibialis anterior left and/or m. tibialis anterior right

*= Switched the DBS off
