# Supplementary material for: Identifying the Diagnostic Challenges and Indicators of Orthostatic Tremor: Patient Perspectives
Source: Mov Disord Clin Pract. 2025 Apr 23;12(8):1124–31. doi: 10.1002/mdc3.70081 (PMC12371454; doi:10.1002/mdc3.70081)
Supplement: Supplementary file 8 — Table S3. Comorbidities. [file MDC3-12-1124-s007.docx]

Table S3. *Comorbidities*.

Values are given either given in absolute numbers (percentage of total).

Abbreviations: n= number of subjects

Abbreviations: EMG= electromyography, n.k.= not known

a= Duration of the disease from diagnosis till inclusion in the study

b= Data recorded from the m. tibialis anterior left and/or m. tibialis anterior right

*= Switched the DBS off

| Disorder | Total (n=360) | Confirmed EMG (n=147) | Reported EMG (n=213) |
| --- | --- | --- | --- |
| Orthostatic tremor only | 213 (59.2%) | 92 (62.6%) | 121 (56.8%) |
| Anxiety | 80 (22.2%) | 29 (19.7%) | 51 (23.9%) |
| Depression | 58 (16.1%) | 22 (15%) | 36 (16.9%) |
| Essential Tremor | 43 (11.9%) | 12 (8.2%) | 31 (14.6%) |
| Restless Legs Syndrome | 17 (4.7%) | 8 (5.4%) | 9 (4.2%) |
| Ataxia | 7 (1.9%) | 2 (1.4%) | 5 (2.3%) |
| Dystonia | 7 (1.9%) | 4 (2.7%) | 3 (1.4%) |
| Parkinson’s Disease/Parkinsonism | 6 (1.7%) | 4 (2.7%) | 2 (0.9%) |
| Orthostatic Myoclonus | 4 (1.1%) | 3 (2%) | 1 (0.5%) |
| Epilepsy | 4 (1.1%) | 2 (1.4%) | 2 (0.9%) |
| Fibromyalgia | 2 (0.6%) | 1 (0.7%) | 1 (0.5%) |
| Dementia | 1 (0.3%) | 1 (0.7%) |  |
| Multiple Sclerosis | 1 (0.3%) |  | 1 (0.5%) |
| Myasthenia Gravis | 1 (0.3%) | 1 (0.7%) |  |
